# Improving the Safety of DIEP Flap Transplantation: Detailed Perforator Anatomy Study Using Preoperative CTA †

**DOI:** 10.3390/jpm12050701

**Published:** 2022-04-28

**Authors:** Katharina Frank, Armin Ströbel, Ingo Ludolph, Theresa Hauck, Matthias S. May, Justus P. Beier, Raymund E. Horch, Andreas Arkudas

**Affiliations:** 1 Laboratory for Tissue Engineering and Regenerative Medicine, Department of Plastic and Hand Surgery, University Hospital Erlangen, Friedrich-Alexander University Erlangen-Nürnberg (FAU), 91054 Erlangen, Germany; frank_katharina@gmx.net (K.F.); ingo.ludolph@uk-erlangen.de (I.L.); theresa.hauck@uk-erlangen.de (T.H.); jbeier@ukaachen.de (J.P.B.); raymund.horch@uk-erlangen.de (R.E.H.); 2 Center for Clinical Studies, University Hospital Erlangen, Friedrich-Alexander University Erlangen-Nürnberg (FAU), 91054 Erlangen, Germany; armin.stroebel@uk-erlangen.de; 3 Department of Radiology, University Hospital Erlangen, Friedrich-Alexander University Erlangen-Nürnberg (FAU), 91054 Erlangen, Germany; matthias.may@uk-erlangen.de; 4 Department of Plastic Surgery, Hand Surgery–Burn Center, University Hospital RWTH Aachen, 52074 Aachen, Germany

**Keywords:** CTA, autologous breast reconstruction, DIEP flap, MS-TRAM flap, perforator

## Abstract

Background: Deep inferior epigastric perforator and muscle sparing transverse rectus abdominis muscle flaps are commonly used flaps for autologous breast reconstruction. CT-angiography allows to analyse the perforator course preoperatively. Our aim was to compare the different aspects of perforator anatomy in the most detailed study. Methods: CT-angiographies of 300 female patients with autologous breast reconstruction of 10 years were analysed regarding the anatomy of the deep inferior epigastric artery and every perforator. Results: Overall, 2260 perforators were included. We identified correlations regarding the DIEA branching point and number of perforators and their intramuscular course. The largest perforator emerged more often from the medial branch of the DIEA than the smaller perforators (70% (416/595) vs. 54% (878/1634), *p* < 0.001) and more often had a direct connection to the SIEV (large 67% (401/595) vs. small 39% (634/1634), *p* < 0.01). Medial row perforators were larger than the laterals (lateral 1.44 mm ± 0.43 (*n* = 941) vs. medial 1.58 mm ± 0.52 (*n* = 1304) (*p* < 0.001)). The larger and more medial the perforator, the more likely it was connected to the SIEV: perforators with direct connection to the SIEV had a diameter of 1.65 mm ± 0.53 (*n* = 1050), perforators with indirect connection had a diameter of 1.43 ± 0.43 (*n* = 1028), perforators without connection had a diameter of 1.31 mm ± 0.37 (*n* = 169) (*p* < 0.001). Medial perforators were more often directly connected to the SIEV than lateral perforators (medial 56% (723/1302) vs. lateral 35% (327/941), *p* < 0.001). A lateral perforator more often had a short intramuscular course than medial perforators (69% (554/800) vs. 45% (474/1055), *p* < 0.001), which was also more often observed in the case of a small perforator and a caudal exit of the rectus sheath. Conclusion: The largest perforator emerges more often from the medial branch of the DIEA and frequently has a direct connection to the SIEV, making medial row perforators ideal for DIEP flap transplantation.

## 1. Introduction

Breast cancer is the most common malignancy among women representing, depending on the literature, approximately 25% of all carcinomas and one of the leading causes of death [1,2,3,4]. If a mastectomy is necessary, breast reconstruction can be performed: implant-based or autologous reconstruction can be performed, the latter one in particular when radiotherapy is part of oncological treatment [5]. Autologous breast reconstruction can be performed using different pedicled or free flaps, which have been improved over the years even up to robot-assisted surgery [6,7,8]. Abdominal-based autologous breast reconstruction using the muscle-sparing transverse rectus abdominis myocutaneous (ms-TRAM), or the deep inferior epigastric perforator flap, is still the method of choice [9,10,11,12,13,14,15].

The perforator vessels are pivotal for a sufficient blood supply of the ms-TRAM/DIEP flap [16,17,18]. For raising such flaps, it is important to know the perforator’s course through the rectus muscle, its characteristics, and any individual anatomy. The perforators originate with different patterns from the deep inferior epigastric artery (DIEA). For the DIEA itself several different branching patterns have also been described. Those aspects need to be collected by the surgeon before surgery so that a safe operation without an intraoperative time delay is possible [19].

Perforator mapping started in 1990 by using Doppler ultrasound [20] and later on by using MRI [21]. The CT angiography (CTA) has been introduced for evaluating these perforator and DIEA parameters prior to surgery in 2006 by Masia [22] and Alonso-Burgos [23] and was confirmed by others like Rozen in 2007 [24] and 2008 [19,25]. The CTA was shown to be superior regarding preoperative ms-TRAM/DIEP flap planning than Doppler ultrasound and MRI [16,19]. This led to better surviving rates of the flaps and a decreased operating time and therefore established CTA as the gold standard [22,23,25,26,27,28,29]. Therefore, preoperative decisions together with different kinds of intra- and postoperative monitoring are pivotal for optimizing postoperative outcome [30].

As an approach to gain a more detailed picture about the anatomic conditions, in this study, the data of 300 patients were collected and analysed especially regarding the number, calibre, course, and anastomosis to the superficial inferior epigastric vein (SIEV) of every single perforator and the branching pattern of the DIEAs. To the best of our knowledge, this is the largest and most detailed study including all perforators detected in the CTAs. 

## 2. Materials and Methods

**Figure 1:**
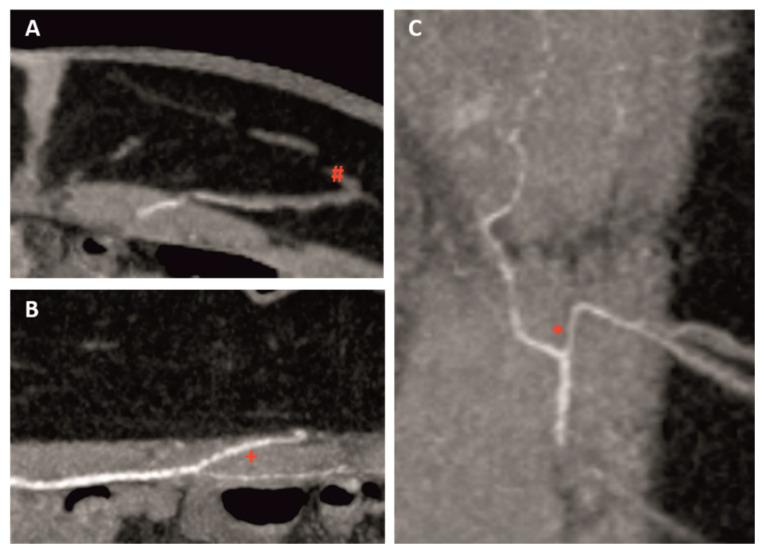
Perforator and DIEA mapping using computed tomographic angiography (CTA) in transversal (**A**), sagittal, (**B**) and coronar (**C**) view (#: connection to SIEV, +: intramuscular course, *: branching of DIEA).

This is a retrospective study including all patients who underwent a single or bilateral autologous breast reconstruction using DIEP or ms-TRAM flaps between January 2010 and October 2019 at the Department of Plastic and Hand Surgery. CTA scans of the abdomen used for other flap planning were excluded. Since 2014, indocyanine green angiography was used for perfusion analysis of DIEP and ms-TRAM flaps. In total, 300 female patients (600 hemiabdominal walls) were evaluated. The patients’ age ranged from 33 to 82 years (mean 63 ± 14 years). The mean body mass index (BMI) was 27.09 kg/m^2^ (range 18.73–40.40 kg/m^2^; SD 4.42). In 43 patients, a bilateral breast reconstruction was performed. Any incidentalomas were registered. All CTA scans were performed at the Radiology Department on a 128 slice multidetector CT (SOMATOM AS+, Siemens Healthcare GmbH, Forchheim, Germany) using a standardized protocol: collimation 64 × 2 × 0.6 mm by z-flying focal spot, rotation time 0.5 sec, spiral pitch factor 0.9, reference tube voltage 120 kV, reference tube current time product 200 mAs. A dose of 60 mL iodine-based contrast medium (Imeron 350, Bracco S.p.A., Milano, Italy) was injected in an antecubital vein in all patients with a flow rate of 5 mL/sec using a power injector (Accutron CT-D, Medtron AG, Saarbrücken, Germany). The CTA scans were reconstructed in thin slices (0.75 mm) with overlapping increments (0.5 mm) for 3D post processing purposes and in overlapping thick slice maximum intensity projections (10 mm/5 mm) in all three planes (transversal, sagittal, coronal) for clinical evaluation (Figure 1). 

The relevant images were analysed from 4 cm above the umbilicus until the symphysis. The level of the umbilicus was always taken as a reference point to determine other parameters such as the perforator’s exit out of the anterior rectus sheet as well as the branching of the DIEA. The localization of any branching or exit point was referred to the umbilicus as the y-axis and the midline for the x-axis. All assessed parameters regarding the DIEA branching, the perforator anatomy, and general parameters are summarized in Table 1. Additionally, the connection to the SIEV was assessed for every perforator and classified as direct, indirect (drainage of superficial fat compartment), or no connection, respectively.

The mean operating time and any inflap anastomosis of the SIEV were noted. Furthermore, the number and DIEA row, i.e., medial vs. lateral row, of perforators included for flap transplantation were evaluated. 

Statistical analysis:

The aims of the statistical analysis were: 1. a descriptive analysis of all collected variables and 2. to search for significant correlation between these variables.

The descriptive analysis used counts, percentages for categorical variables, and mean, median, and standard deviation for interval-scaled variables. 

Correlation was evaluated by bivariate tests of all pairs of variables and subsequent post hoc tests with a correction for multiple testing. Software was GraphPad Prism 8 (GraphPad Software, San Diego, CA, USA) and R 3.6.1 (R Core Team (2019).

## 3. Results

In total, 128 DIEP and 214 ms-TRAM flaps were performed, including 43 bilateral breast reconstructions. Out of those 214 ms-TRAM flaps, there were 2 ms0-, 164 ms1-, and 48 ms2-TRAM flaps. An amount of 600 hemiabdominal walls were analysed with 2260 perforators in total. The correlation of the thickness of the subcutaneous fat (mean 3.0 cm, SD 0.94 cm) and the flap choice indicated that ms-TRAM flaps were more often performed at higher BMIs. 

In half of the cases (48.8%), there was a type II branching according to the Moon and Taylor Classification (modified by Rozen et al. [31]), followed by type I (37.8%), type III (12.7%), and IV (0.5%). Comparing the left to the right hemiabdomen there was a symmetry in most cases. Looking at the most common type II, the average branching point on the x-axis was 4.34 cm lateral and 5.73 cm on the y-axis caudal to the umbilicus. Only in ten cases was the branching cranial to the level of the umbilicus. Another significant observation was the correlation between branching type and the number of perforators: the higher the number of main DIEA branches, the more perforators were detected (correlation, r = 0.2743; *p* < 0.0001).

### 3.1. Diameter and Branching of Perforators 

There were four perforators on each side of the abdomen on average (range 0–11; SD 2) with a diameter of 1.5 mm (SD 0.5 mm). 

An amount of 58% of all perforators emerged off the medial branch of the DIEA and 42% were lateral branch perforators. The average entrance point of the rectus sheath was 3.5 cm lateral (SD 1.9 cm) and 0.94 cm caudal (SD 3.1 cm) of the umbilicus. 

**Figure 2:**
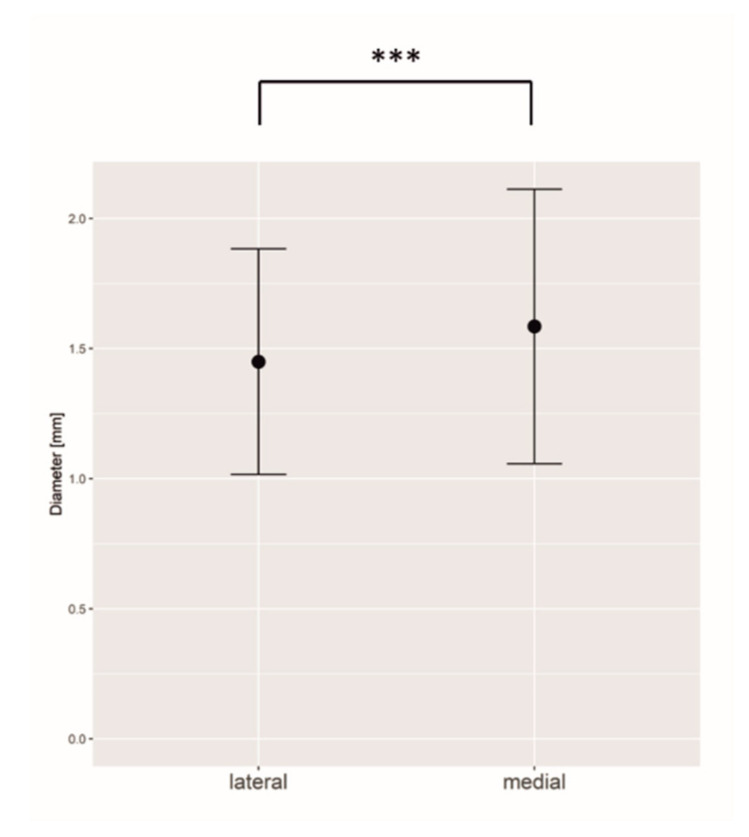
Medial row perforators have a statistically significantly larger diameter than lateral row perforators. *** = *p* ≤ 0.001.

Statistical analysis showed that the diameter of medial row perforators (mean 1.58 mm; SD 0.52 mm) was significantly larger than the diameter of lateral row perforators (mean 1.44 mm; SD 0.43 mm) (*p* < 0.001) (Figure 2).

### 3.2. SIEV

**Figure 3:**
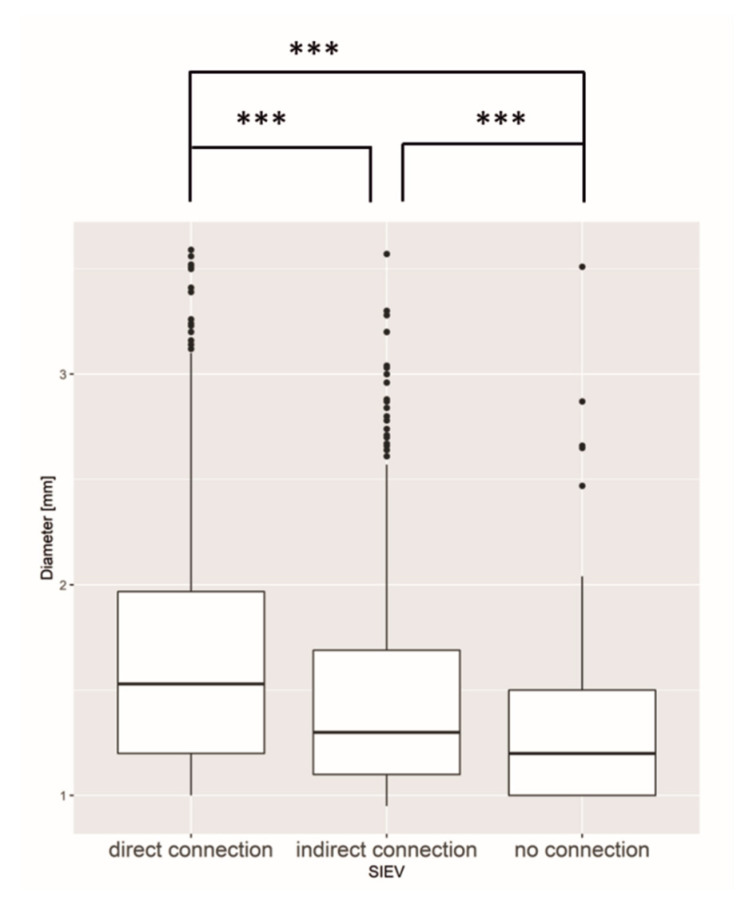
Boxplot of the diameter and different SIEV connections. SIEV = superficial inferior epigastric vein. The diameter of the perforator had a significant influence on the connection to the SIEV. The different connection types varied significantly among themselves. *** = *p* ≤ 0.001.

There was a significant correlation between perforator diameter and SIEV connection. The larger the diameter of the perforator, the more likely a perforator had a connection to the SIEV or the superficial fat compartment (Figure 3). 

**Figure 4:**
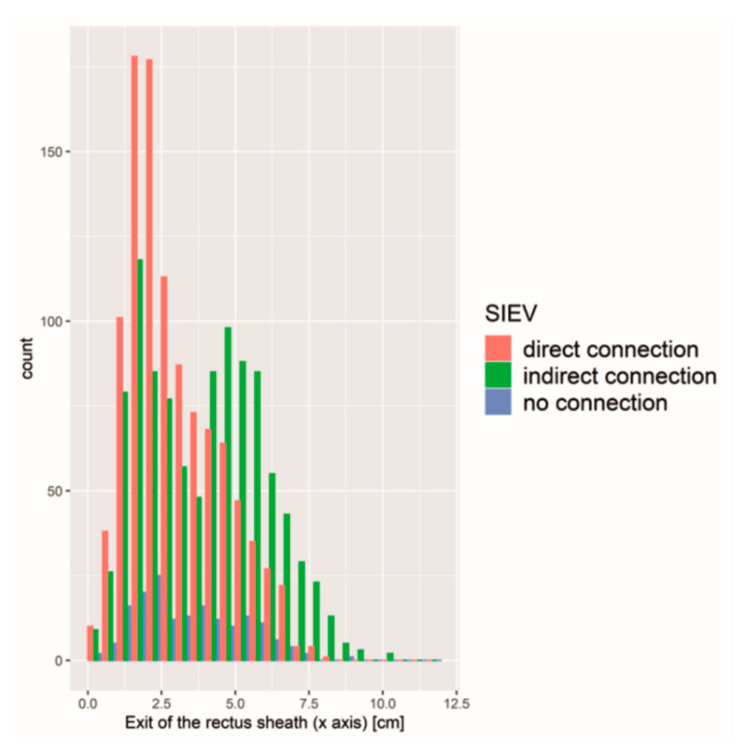
Absolute counts of different SIEV connection types regarding exit of the recuts sheath. Perforators close to the umbilicus were more likely connected to the SIEV.

Next to the diameter, the perforator exit of the rectus sheath on the x- and y-axis had a significant influence on SIEV connectivity (*p* < 0.01). A perforator with a close exit of the rectus sheath to the umbilicus on the x- and y-axis was more likely connected to the SIEV (Figure 4). 

**Figure 5:**
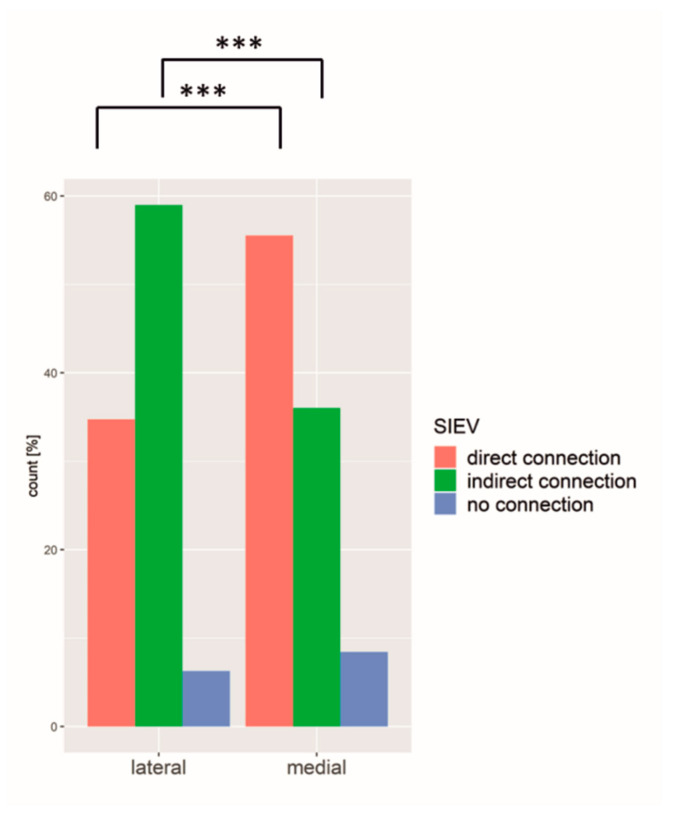
Medial row perforators were more often directly connected to the SIEV compared to lateral perforators. *** = *p* ≤ 0.001.

An amount of 46.9% of the perforators had a direct connection to the SIEV, 45.3% drained only the superficial fat compartment, whereas only 7.7% had no connection to the SIEV or superficial fat compartment at all. The medial row perforator more often showed a direct connection to the SIEV, whereas the lateral row perforator mainly drained the superficial compartment without direct connection to the SIEV (*p* < 0.001) (Figure 5).

### 3.3. Intramuscular Course 

The intramuscular course of each perforator was categorized into “short” (<1.5 cm), “long” (>1.5 cm), and “no intramuscular course” (medially around the rectus muscle) regarding their cranial/caudal course through the rectus abdominis muscle. A total of 45.5% of all perforators showed a short course through the muscle, only 32.4% had a long course, and in only 100 perforators (4.5%) was a course medial around the muscle observed. In 17.7% of all perforators, there was no intramuscular course verifiable, thus making a classification not possible. 

**Figure 6:**
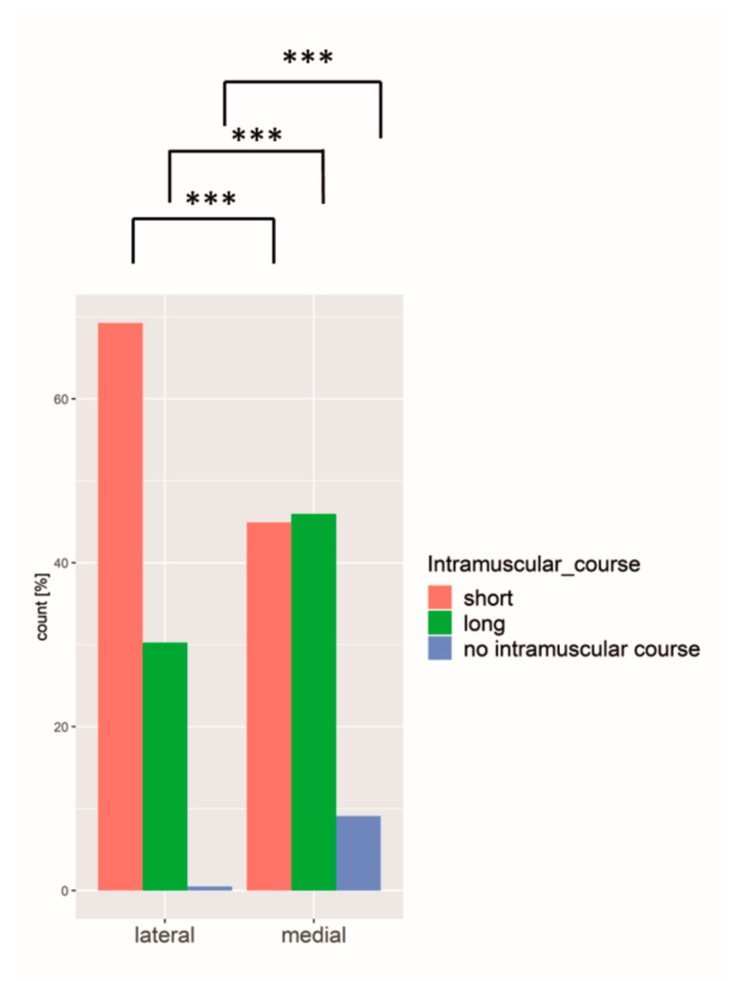
Medial row perforators had a more equally distributed intramuscular course, whereas lateral perforators more often had a short intramuscular course. *** = *p* ≤ 0.001.

Comparing the medial and the lateral row perforators with regard to their intramuscular course, there was a significantly higher number of perforators with short course among the lateral row perforators as compared to medial row perforators (Figure 6). 

Also, the smaller the perforator in diameter, the less likely it had a long intramuscular course (one-way ANOVA, *p* < 0.005). 

Furthermore, the course medially around the rectus muscle (also termed “septal”, i.e., no intramuscular course at all) applied to 2/3 of the large perforators and to 1/3 of the small perforators. 

### 3.4. Largest Perforator (No 1) 

The largest perforator of every hemiabdomen was classified as the perforator no 1. 

**Figure 7:**
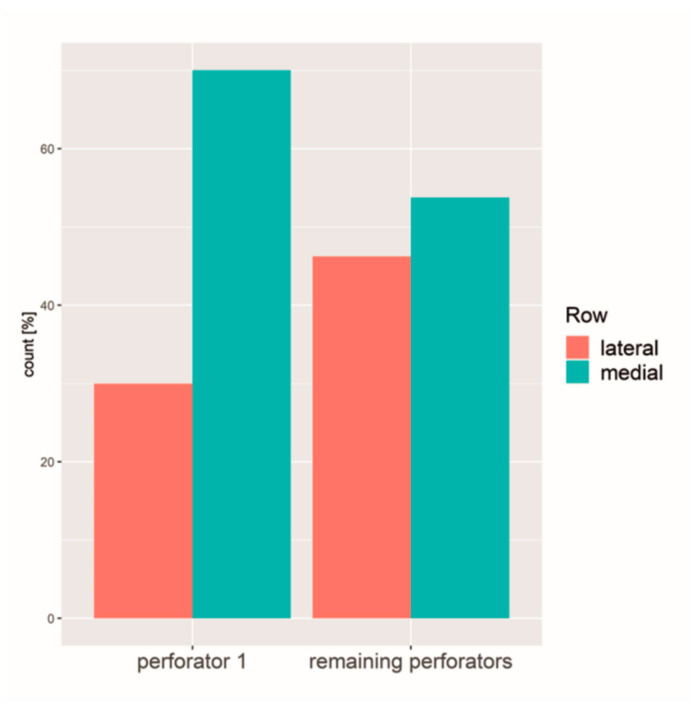
Distribution of the largest perforator versus the remaining perforators regarding the branch of the DIEA.

**Figure 8:**
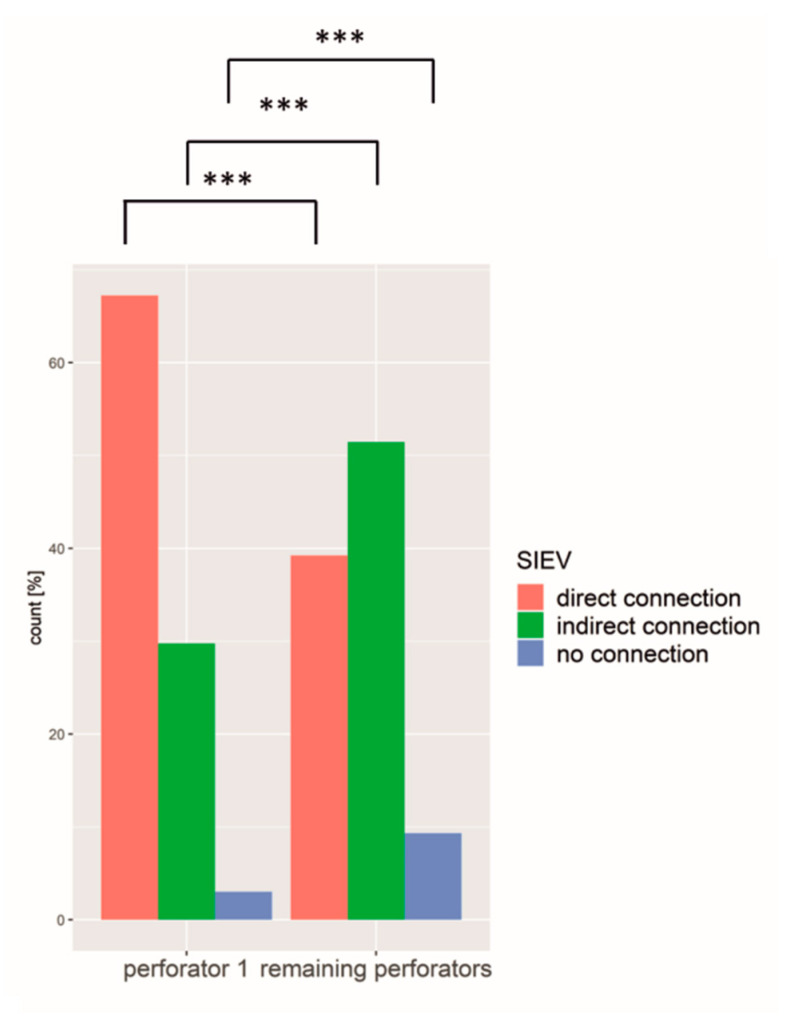
The largest perforator was significantly more often directly connected to the SIEV compared to the remaining perforators, whereas the remaining perforators were more often indirectly connected or without any connection. *** = *p* ≤ 0.001.

Comparing the perforator no 1 with a mean diameter of 2.0 mm (range 1–3.59 mm, SD 0.56 mm) with the remaining perforators (mean diameter of 1.4 mm, range 0.95–3.1 mm, SD 0.35 mm), it differed in all variables significantly. In 70%, the largest perforator originated from the medial branch of the DIEA, whereas the rest had a uniform distribution of the medial and lateral branch (Figure 7) and exited closer to the umbilicus. Furthermore, perforator no 1 was more often directly connected to the SIEV compared to the other perforators (Figure 8). 

## 4. Discussion

CTA has become the gold standard in preoperative diagnosis prior to autologous breast reconstruction using DIEP or ms-TRAM flaps [23] even though there is the disadvantage of radiation and higher costs compared to the Doppler ultrasound [32]. By knowing the course and characteristic of the perforators preoperatively, it allows the surgeon to reduce operating time and increase the safety of the operation. It also helps to decide whether a DIEP or ms-TRAM flap will be raised. In this retrospective study, we analysed every single perforator in 600 hemiabdominal walls to gain further insight into the perforators’ characteristics and anatomy. 

The main findings were that the largest perforator emerges in 2/3 of the medial row. Additionally, medial row perforators have a larger diameter than lateral row perforators. Furthermore, the closer the perforator leaves the rectus sheath to the umbilicus, the larger was the diameter. Additionally, medial row perforators are more likely connected to the SIEV than lateral row perforators. Regarding the perforator no 1, it differs significantly from the remaining perforators. The mean diameter of perforator no 1 was 2.0 mm compared to a diameter of 1.5 mm for perforator no 2, and 1.3 mm for perforator no 3. Schrögendorfer et al. noticed a mean diameter of 1.73 mm without splitting into different perforators [33]. In Masia et al., the dominant perforator’s diameter ranged from 0.6 to 3.2 mm [34], which is similar to our range of 1–3.6 mm. Furthermore, perforator no 1 was almost twice as much as the remaining perforators connected to the SIEV, it was more equally distributed concerning short and long intramuscular course, it left the rectus sheath mainly medially, thus closer to the umbilicus on both axes. Therefore, we used a coordinate system that was already established in other studies [26,35,36]. Mohan et al. also detected that the larger dominant perforators arise mainly from the medial row around the umbilicus, calling it the “hot spot” [37]. Analysing the vascular territories using adult cadavers, Wong et al. even broke it down by comparing different flap types. The perfusion was found to be the largest in the full-width TRAM flap followed by the almost comparable medial row-perfused ms-TRAM and the medial row perforator DIEP flap [38].

Using the modified Moon and Taylor classification for grading the DIEA in a coronal view, we found a type II branching in 49%. The Moon and Taylor study showed 57% type II DIEAs, whereas Pellegrin only noticed a type II in 28% and mainly type I (65%) [39]. Masia et al. also included over 300 patients, but they analysed the perforators for only some patients in greater detail [34]. Regarding the branching pattern, they mainly detected a type II (right hemiabdomen: 58.8%, left hemiabdomen: 52.8%) and the rest showed a type I branching pattern. Rozen et al. showed a similar distribution to ours (I 29%, II 57%, III 14%). Masia’s caudal point of the branching pattern was also closer to the umbilicus (5.05 cm) compared to our branching point, which was 5.73 cm caudal of the umbilicus. The Navarra conference led to the consensus that analysing the branching pattern in an anterior view is very helpful [32]. Almost every single branching point was caudal from the level of the umbilicus. We were able to show that the more branchings of the DIEA, the more perforators were detectable. Having four perforators on each side, the mean diameter for the largest perforator was 1.9 mm on the left and 2 mm on the right side [35]. Pellegrin et al. found fewer perforators [39] and differences between the left and the right side: three perforating branches on the right and two on the left side. This might be due to different inclusion criteria since in our study every single perforator that was found was analysed. 

We also found that the closer the exit of the rectus sheath to the umbilicus on the x-axis, the larger the diameter of the largest perforator. Bailey et al. already confirmed this in a smaller study only analysing the largest perforator [26]. In several studies, it was also observed that those perforators that are located periumbilical show better perfusion of the elevated flaps [40,41]. While we decided to classify the cranial/caudal intramuscular course as “short” for distances under 1.5 cm, we found that mainly the lateral perforators more often had a short intramuscular course compared to a long intramuscular course. This was also detected by Rozen et al. in two studies but with fewer patients [25,29].

There is no standard yet for how to classify the intramuscular course and there are various options, whereas ours is only one of them. Navarra et al. analysed the intramuscular course in an axial MIP view [32]. This might lead to difficulties in comparing different studies. Comparing medial with lateral branch perforators, we found a distribution of 58% medial perforators versus 42% lateral perforators and significantly larger medial than lateral branch perforators. Similar results in Kukrek et al. confirm the occurrence of more medial perforators [42]. In their analysis, almost 50% of the perforators had a long intramuscular course, only 39% had a short intramuscular course, and 11% had a course medial around the rectus muscle, whereas in our study, the short course was predominate with 45%. This difference might depend on a different classification of the intramuscular course and fewer patients included in their study compared to ours. 

The SIEV is an often undervalued topic in other studies, hence we analysed the connection of every perforator to the SIEV and found out that medial row perforators more often have a direct connection, whereas lateral row perforators often do not have a direct connection but drain the superficial fat compartment. Similarly, during the Navarra meeting, they found consensus that the venous system plays an immense role [32]. This was also mentioned by Zhang et al. without dealing in detail with the SIEV communicating with the perforators [43]. Between the right and left hemiabdomen, we did not find any statistically significant difference, thus it can be considered as symmetrically distributed. In this study, we have not evaluated the SIEA anatomy due to the focus on the DIEA anatomy and the arising perforators. We also did not correlate the CTA findings with intraoperative perforator choice and diameter and ICG flap viability due to the retrospective character of the study.

Bailey et al. compared the dominant medial row perforators on the right and left side and noticed symmetry as well [26]. Number and diameter were analysed in Rosson et al. and also identified as symmetric [28]. 

With a mean BMI of 27 kg/m^2^, most of our patients were overweight. This matches the observations seen in other studies. Zang et al. had a smaller mean BMI of 23.6 kg/m^2^ probably resulting from the fact that only Asian women were included [43]. Furthermore, the higher the BMI, the more likely a perforator emerges more lateral through the rectus sheath. A reason for this might also be a potential rectus diastase. 

We acknowledge some limitations of our study. Due to the high number of perforators (>2000), pairwise statistical tests showed a significant result for almost everything irrespective of the strength of their connection. The enhanced artery and concomitant unenhanced veins run close together and are sometimes hard to distinguish making measurement of the perforator diameter difficult. Whereas this study dealt with the medical aspects concerning optimizing operative outcome, it is also important to consider the patient’s perspective to gain the optimal result, for example, by using the BREAST-Q assessment [44,45,46]. Due to the retrospective character of the study, no correlation of the CTA findings with intraoperative findings was possible and therefore no outcome analysis like Breast-Q assessment was performed.

To the best of our knowledge, this is the largest anatomical study of CTAs prior autologous breast reconstruction. 

## 5. Conclusions

The preoperative CTA provides multiple information regarding perforator anatomy, which were analysed in this study in great detail. We were able to show that the largest perforator emerges more often from the medial branch of the DIEA and more often has a direct connection to the SIEV. Large perforators were most likely found around the umbilicus and arising from the medial row. Additionally, medial row perforators showed more direct connections to the SIEV and larger diameters compared to lateral row perforators, making them ideal for DIEP flap transplantation.

## Figures and Tables

**Table 1:** Assessed parameters of CTAs.

**DIEA Branching**	
Branching pattern	Type 0-IV according to Moon and Taylor classification (modified by Rozen et al.)
Branching pattern point	Localization on the x- and y-axis
**Perforator**	Sorted by diameter
Diameter	At the exit of the rectus sheath
Entrance of the perforator into the DIEA branch	Localization on the x- and y-axis
	Medial/lateral
Intramuscular course	Short (<1.5 cm), long (>1.5 cm), no intramuscular course (medially around the rectus muscle)
Exit of the rectus sheath	Localization on the x- and y-axis
SIEV	Direct connection, indirect connection, no connection to upper fat compartment
**Subcutaneous fat**	Thickness 3 cm to the right and left of the umbilicus
**General information**	- Operating time - Incidentalomas - Intraoperative SIEV anastomosis if necessary - flap type - Intraoperative used perforators - BMI/height/weight

## Data Availability

Not applicable.
